# *Prevotella bivia* promotes cervical cancer progression and modulates macrophage polarization, while *Lactobacillus iners* suppresses these processes: evidence from multiomics analysis

**DOI:** 10.1128/mbio.00374-26

**Published:** 2026-06-15

**Authors:** Ting Zhang, Qiuhong Qian, Qianwei Zhen, Jiaxin Zhang, Yu Sun, Junhua Zhang, Lingyu Guo, Xiaoli Liu, Deyuan Zou, Baokun Zhou, Chang Liu, Shuqi Chi, Gaishuang Shang, Baoxia Cui, Youzhong Zhang, Yunlong Cui, Youming Zhang, Sai Han

**Affiliations:** 1 Department of Obstetrics and Gynecology, Qilu Hospital of Shandong University 91623, Jinan, Shandong, People's Republic of China; 2 Shandong Key Laboratory of Reproductive Health and Birth Defects Prevention and Control, Shandong University, Jinan, Shandong, People's Republic of China; 3 The Second Qilu Hospital of Shandong University 531675, Jinan, Shandong, People's Republic of China; 4 Eastsea Pharma Co. Ltd, Qingdao, Shandong, People's Republic of China; 5 State Key Laboratory of Microbial Technology, Shandong University, Qingdao, Shandong, People's Republic of China; Columbia University, New York, New York, USA

**Keywords:** cervical cancer, intratumoral microbiome, *Lactobacillus iners*/*Prevotella bivia*, macrophage, 16S rRNA

## Abstract

The incidence and mortality rates of cervical cancer are high among women. Growing evidence suggests the key roles of the intratumor microbiome in various solid tumors. However, the intratumoral microbiome in patients with cervical cancer has not been well characterized. In this study, 16S rRNA sequencing was performed on 76 tissues to reveal the features of the intratumoral microbiome, and the highly differentially abundant bacteria *Lactobacillus iners*/*Prevotella bivia* were selected for functional verification. Flow cytometry, transwell migration, and invasion experiments, among others, were performed *in vitro*; subcutaneous tumor formation and lung metastasis experiments were performed *in vivo*. Additionally, macrophages were cocultured with the *L. iners*/*P. bivia* supernatant to evaluate how the intratumoral microbiome affects their polarization, and tumor cell and macrophage transcriptome sequencing was subsequently performed to explore the potential molecular mechanisms involved. Metabolomic analysis of tissues and bacterial supernatants was performed to search for potential carcinogens and cancer suppressors. We determined that the abundance of microbes was greater in cervical cancer tissues than in normal cervical and paracancerous tissues. The relative abundance of *Prevotella* was correlated with deep stromal invasion, tumor size, clinical stage, and poor survival prognosis in cervical cancer patients. *L. iners* inhibited the proliferation and promoted the apoptosis of tumor cells, whereas *P. bivia* significantly promoted cervical cancer cell migration and invasion. Mechanistically, the modulation of phosphorylated PI3K/AKT/mTOR signaling may be involved in the observed effects. *P. bivia* promoted M2 macrophage polarization by activating phosphorylated STAT3 and NF-κB, but the role of *L. iners* remains unknown. The amino acid metabolism, carbohydrate metabolism, and lipid metabolism pathways were enriched in differentially abundant metabolites such as glycine, which may be a key molecule. This study provides evidence that the intratumoral microbiome, represented by *L. iners*/*P. bivia*, affects tumor biology and macrophage polarization in patients with cervical cancer.

**IMPORTANCE:**

The microbiome in cervical cancer tissues significantly differed from that in normal cervical tissues and showed significant correlations with clinicopathological features and survival prognosis. The tumor microbiome affects the biological behavior of cervical tumor cells and the polarization of macrophages through metabolite production, thus playing an important role in the occurrence and development of cervical cancer.

## INTRODUCTION

Cervical cancer remains the most prevalent gynecological malignancy and a leading cause of cancer-associated mortality in women worldwide. According to recent WHO data, approximately 600,000 new cases and 340,000 deaths were reported globally in 2022, and the disease burden was particularly high in China (1, 2). The persistent challenges in eliminating cervical cancer cells highlight the urgent need for new studies to advance the understanding of its pathogenesis and identify new potential avenues for intervention.

In recent years, accumulating evidence has established the presence of distinct intratumoral microbiomes across a variety of solid tumors—including those of the colon, breast, lung, and pancreas; studies have also revealed that tumor-resident bacteria play key roles in modulating tumor progression, metastasis, prognosis, and even treatment response (3–6). For example, in breast tumors, unique bacteria such as *Staphylococcus* and *Lactobacillus* drive metastatic colonization (4), and the presence of *Fusobacterium* in the tumor microenvironment of colorectal cancers is correlated with poor survival and altered immune infiltration (6). Mechanistic studies indicate that these bacteria may influence cancer biology through the modulation of tumor metabolism, immune responses, and cell signaling pathways.

Despite such progress in other malignancies, the significance and characteristics of the intratumoral microbiome remain largely undefined in the context of cervical cancer. Recent studies have focused on cervical and vaginal microbial communities in the context of HPV infection, a well-established etiological factor in cervical carcinogenesis, and have revealed shifts in the vaginal microbiome that are associated with disease progression (7). However, few investigations have directly addressed the microbiome composition within cervical tumor tissues, its differences from that in adjacent or normal cervical tissues, or its impact on tumor biology, immune cell function, and clinical outcomes. Furthermore, the potential interplay between the tumor microbiome and key factors unique to cervical cancer, such as persistent HPV infection, remains unexplored.

Given the diversity and tumor-type specificity of intratumoral bacteria revealed in other cancers, determining whether cervical cancers harbor a unique microbiome that may shape their progression through distinct mechanisms is critical. This is a particularly important task in cervical cancer research, given the unique dependence of this cancer on HPV-driven oncogenesis and the increasing recognition of the tumor immune microenvironment—including the role of tumor-associated macrophages in immune escape (8). The possibility that tumor-resident bacteria interact with these cervical cancer-specific factors to modulate disease progression, immune evasion, and prognosis represents an important but unresolved theory in cervical cancer research.

In this study, we performed 16S rRNA sequencing on tumor and adjacent tissues from cervical cancer patients and normal cervical tissues and used computational biology methods to quantify the bacteria in the tissues and analyze their relationships with the clinicopathological features of cervical cancer patients. We also confirmed the presence of intracellular bacteria in cervical cancer tissues and adjacent tissues using tissue microarrays and an EdU probe. Furthermore, we demonstrated the biological functions of intratumoral bacteria in cervical cancer *in vitro* and *in vivo* and analyzed the impact of intratumoral bacteria on macrophages in a coculture model. Finally, we studied the potential mechanisms involved via transcriptomic and metabolomic approaches. In summary, our research provides a comprehensive overview of the intratumoral microbiome in patients with cervical cancer and investigates its role in the occurrence and development of this disease.

## MATERIALS AND METHODS

### Patient selection and sample collection

Tissue samples were collected from 23 normal controls and 40 cervical cancer patients (13 of whom had paracancerous tissues) admitted to the Department of Obstetrics and Gynecology, Qilu Hospital of Shandong University, from 2018 to 2021. All clinical data, including age and pathological diagnosis, were collected, and the patients were staged according to the 2018 FIGO staging guidelines. The present study was approved by the Ethics Committee of Qilu Hospital. In this study, all human tumor and normal cervical tissue samples were freshly collected during surgery. Immediately after excision, the samples were placed into sterile tubes and rapidly snap-frozen in liquid nitrogen to preserve microbial DNA integrity and minimize potential contamination. Formalin-fixed paraffin-embedded (FFPE) samples were not used.

### 16S rRNA sequencing and bioinformatics analysis

During sample processing and DNA extraction, strict aseptic techniques were employed using sterilized instruments and consumables under sterile conditions to prevent contamination. Negative controls, such as negative control samples, were incorporated during the PCR amplification step, and nuclease-free water provided in commercial nucleic acid extraction kits was used as a template to monitor potential contamination during PCR. Genomic DNA was extracted from the tumor samples using hexadecyl trimethyl ammonium bromide (CTAB). The rDNA of the bacterial ribosome subunit 16S (V4 region) was amplified using the universal primers 515 F (5′-GTGCCAGCMGCCGCGGTAA-3′) and 806 R (5′-GGACTACHVGGGTWTCTAAT-3′). After the samples were compiled, inspected for quality, quantified, and mixed in equal proportions, they were sequenced using the NovaSeq 6000 platform (Illumina, San Diego, CA, USA). The paired-end reads were subsequently merged using FLASH (V1.2.11, http://ccb.jhu.edu/software/FLASH/), and quality filtering was performed using fastp software (version 0.23.1) to obtain high-quality clean reads (9, 10). For the effective reads obtained previously, denoising was performed with DADA2 or the deblur module in QIIME2 software (version QIIME2-202202) to obtain the initial amplicon sequence variants (ASVs) (DADA2) (11). The top axis of each sample at each taxonomic rank (phylum, class, order, family, genus, and species) was used to plot a distribution histogram of relative abundance using the SVG function in Perl. The abundances of the top taxa of each sample at each taxonomic rank were used to construct a heatmap. Venn diagrams were produced in R with the VennDiagram() function. The 100 most abundant genera were subjected to sequence alignment, and a phylogenetic tree was constructed using the SVG function in Perl. The alpha diversity was calculated from seven indices, observed_otus, Chao1, Shannon, Simpson, dominance, Good’s coverage, and Pielou_e, using QIIME2. To evaluate the complexity of the community composition and evaluate the differences among samples (groups), beta diversity was calculated on the basis of weighted and unweighted UniFrac distances using QIIME2. A cluster tree was constructed on an unweighted pair group arithmetic average (UPGMA) diagram using the upgma.tre function within QIIME2. Nonmetric multidimensional scaling (NMDS) was implemented with the ade4 and ggplot2 R packages. Differences in community structure were revealed via ANOSIM, Adonis, the multiresponse permutation procedure (MRPP), SIMPER, *t* tests, metagenomeSeq, and linear discriminant analysis (LDA) effect size (LEfSe) analyses. Anosim, Adonis, MRPP, and SIMPER analyses were performed with the vegan and ggplot2 packages within R; metagenomeSeq was performed with the metagenomeSeq package in R; and the exclusive package lefse was utilized for LEfSe. For the LDA and LEfSe analyses, the power comparison control strategy was set as one against one, the LDA threshold was set to 4, and the classification level threshold was set to 0.05 (12).

### Transcriptome sequencing and bioinformatics analysis

RNA integrity was assessed using an RNA Nano 6000 Assay Kit and a Bioanalyzer 2100 system (Agilent Technologies, CA, USA). After library preparation, clustering and transcriptome sequencing were performed. Clustering of the index-coded samples was performed on a cBot Cluster Generation system using a TruSeq PE Cluster Kit v3-cBot-HS (Illumina) according to the manufacturer’s instructions. The generated clusters and library preparations were subsequently sequenced on an Illumina NovaSeq platform, and 150 bp paired-end reads were generated. Analysis of the differentially expressed factors between the two groups was performed using the DESeq2 package in R (1.20.0). The obtained *P* values were adjusted using Benjamini and Hochberg’s approach to control the false discovery rate. Genes with an adjusted *P* value ≤0.05 as determined by DESeq2 were considered differentially expressed. We then used the clusterProfiler package in R to evaluate the pathways with significant enrichment of differentially expressed genes using the Kyoto Encyclopedia of Genes and Genomes (KEGG) database (http://www.genome.jp/kegg/).

### Metabolomic sequencing and bioinformatics analysis

A nontargeted metabolomics study was conducted using a liquid chromatography-mass spectrometer (LC-MS/MS) (13, 14). For these experiments, the metabolites were extracted from the samples and subjected to LC-MS/MS and data analysis. Three main parameters were considered when screening differentially abundant metabolites: the variable importance in projection (VIP), the fold change (FC), and the *P* value. The VIP refers to the first principal component from the partial least squares discriminant analysis (PLS-DA) model and represents the contribution of the metabolites to the clustering; the FC was calculated as the ratio of the mean value of all quantified metabolites from each replicate in the comparison group, and the *P* value, which indicates the significance of the differences among the data, was calculated by a *t* test. The thresholds for the differentially abundant metabolites were a VIP >1.0, FC >1.2 or an FC <0.833 and a *P* value <0.05. The identified metabolites were subjected to functional annotation using mainly the KEGG database, as mentioned above.

### Cell lines and culture

The human cervical cell lines HeLa and SiHa, and the monocytic THP-1 cell line, which were purchased from the Cell Bank of the Chinese Academy of Sciences (Shanghai, China), were used in this study. The cells were maintained in RPMI-1640 medium supplemented with 10% fetal bovine serum (FBS; Biological Industries, USA) and incubated under standard culture conditions (5% CO_2_, 37°C).

### Extraction of bacterial supernatant

In this study, bacterial culture supernatants from both *in vitro* and *in vivo* experiments were sterile-filtered through 0.22 µm membranes to remove all viable bacteria. The supernatants were subsequently adjusted with sterile phosphate-buffered saline (PBS) to achieve physiological osmolarity (~290 mOsm/kg) and a neutral pH (~7.4) to minimize nonspecific effects. Given that *Prevotella bivia* is a gram-negative bacterium capable of releasing lipopolysaccharides (LPS), we acknowledged that residual endotoxins might induce cellular stress responses and potentially confound data interpretation. Therefore, endotoxin levels in the collected supernatants were quantified using the limulus amebocyte lysate (LAL) assay, which revealed that endotoxin concentrations were below the thresholds commonly recognized to significantly affect mammalian cells.

### Flow cytometry

To detect apoptosis and the cell cycle, we established three groups of cell samples: control cells, SiHa cells treated with *Lactobacillus* and *Prevotella* supernatants, and HeLa cells treated with the same supernatants. Apoptosis: The control group and the SiHa and HeLa cells treated with *Lactobacillus* and *Prevotella* supernatants were digested using trypsin without EDTA. Next, the cells were stained with an Annexin V-FITC/PI Apoptosis Kit (Elabscience, Wuhan, China) for 30 minutes at room temperature in the dark. Cell cycle: The cells were digested with trypsin containing EDTA and then fixed in 75% ethanol. After overnight fixation, PI/RNase Staining Buffer (BD Biosciences, Franklin Lakes, NJ, USA) was added to the respective fixed samples. The samples were incubated for 15 minutes at room temperature in the dark. Apoptosis and the cell cycle were detected by flow cytometry (Cytoflex, USA), and quantitative analysis was performed using FlowJo v10 and CytExpert.

### Transwell migration and invasion assays

After coculture, 5 × 10^4^ SiHa or HeLa cells were digested, suspended in 100 µL of serum-free 1640 medium, and seeded into the upper chamber of a Transwell system (8.0-µm pore size; Costar, Cambridge, MA, USA) in the absence or presence of 100 µL of Matrigel (diluted 1:8 in serum-free medium; Corning, NY, USA). The experiments were performed as previously described (15).

### Reverse transcription real-time PCR

Cellular RNA was extracted using a SPAReasy cell RNA rapid extraction kit (SparkJade, Jinan, Shandong, China), and cDNA was synthesized from 5 µg of RNA using M-MLV reverse transcriptase (Invitrogen, Shanghai, China) according to the manufacturer’s instructions. In addition, primer–probe sets were designed for each gene for qPCR using PrimerBank and are listed in Table S1. The data were collected using the StepOnePlus software (Applied Biosystems, Shanghai, China) and quantified using the 2^−ΔΔCt^ method.

### Western blotting

Total protein was extracted from the cells as previously described (15). The primary antibodies used were anti-p-PI3K (1:1,000; Cell Signaling Technology, #4228T), anti-p-AKT (1:1,000; Cell Signaling Technology, #4060S), anti-p-mTOR (1:1,000; Cell Signaling Technology, #2971S), anti-p-4E-BP1 (1:1,000; Cell Signaling Technology, #2855T), anti-p70 S6 kinase (49D7) (1:1,000; Cell Signaling Technology, #2708T), anti-p-STAT3 (1:1,000; Abcam, ab46154), anti-p-NF-κB (1:1,000; Cell Signaling Technology, #3033T), anti-IL-6 (1:1,000; Immunoway, #YT5348), and anti-β-actin (1:80,000; ABclonal, #AC026); notably, β-actin is considered a very stable internal control. The secondary antibodies used were anti-rabbit (1:5,000; Immunoway, #RS0002) and anti-mouse (1:2,000; Cell Signaling Technology, 7076S) IgG peroxidase conjugates. A Tanon 4800 multifunctional imaging system (Tanon Life Science Co., Ltd., Shanghai, China) was used for protein detection, and the results were analyzed with ImageJ software (NIH, Bethesda, MD, USA).

### Tumor cell growth and metastasis in athymic nude mice

Twelve 4-week-old female athymic nude mice purchased from Weitong Lihua Biotechnology Co., Ltd. (Beijing, China) were used in this study and randomly divided into three groups (4 mice/group). Upon reaching 80% confluence, the HeLa cells were digested with 0.25% trypsin and collected by centrifugation at 1,000 rpm for 5 min. A cell suspension with a concentration of 5 × 10^6^ cells/mL was then subcutaneously injected into the mice. The tumor volume and size were measured as previously described (16, 17). Additionally, the supernatants of the two test species and phosphate-buffered saline (PBS) were injected into the abdominal cavity every 2 days. After 18 days, the xenografts were removed from the mice and photographed. The lung metastasis model also included 12 female athymic nude mice randomly divided into three groups (4 mice/group). For these experiments, HeLa cells (1 × 10^6^ cells/mL) were injected into the mice through the tail vein. The mice received injections of the supernatant of the two test bacterial species and PBS for two months, after which the lungs were removed for hematoxylin and eosin (HE) staining to identify tumor nodules.

### Statistical analysis

Statistical analyses were conducted with GraphPad Prism 8.0 software (GraphPad Software Inc., San Diego, CA, USA). All the experiments were repeated at least three times, and the results are presented as the means  ±  standard deviations (SDs). Student’s *t* test, the Pearson *r* test, the chi-square test, and Yate’s continuity-corrected chi-square test were performed to analyze the data from the different groups. Finally, the Kaplan–Meier method and the log-rank test were used to analyze the survival data. *P*  <  0.05 was considered to indicate statistical significance.

## RESULTS

### Patients

We collected 76 clinical samples from patients (40 cervical cancer tissues [C] plus 13 paracancerous tissues [Np] and 23 normal cervical tissues [Nc]). The clinicopathological features of the cervical cancer patients are presented in Table 1.

**TABLE 1:** Association between Lactobacillus/Prevotella relative abundance and the clinicopathological factors^*a*^

Variable	No.	Expression of *Lactobacillus*			Expression of *Prevotella*		
		High	Low	*P*-value	High	Low	*P*-value^*d*^
Age				0.7357			0.7357
≤45	13	7	6		7	6	
>45	27	13	14		13	14	
Histology				0.7150			1.000
Adenocarcinoma	10	6	4		5	5	
SCC	30	14	16		15	15	
Differentiation				1.000			0.5186
Low	16	8	8		7	9	
Moderate/high	24	12	12		13	11	
Clinical stage				0.1769			**0.0428^*b*^**
≥IB3	27	11	16		17	10	
<IB3	13	9	4		3	10	
Tumor size				0.5250			**0.0110^*b*^**
<4 cm	22	10	12		7	15	
≥4 cm	18	10	8		13	5	
LVSI				0.5073			1.000
Negative	14	8	6		7	7	
Positive	26	12	14		13	13	
LNM				1.000			0.1848
Negative	26	13	13		11	15	
Positive	14	7	7		9	5	
DSI				0.7233			**0.0046^*c*^**
<1/2	11	6	5		1	10	
≥1/2	29	14	15		19	10	

^*a*^ SCC, squamous cell carcinoma; LNM, lymph node metastasis; LVSI, lymph vascular space involvement; DSI, deep interstitial infiltration, using Pearson’s correlation analysis.

^*b*^ Statistically significant value. *P* < 0.05.

^*c*^ Statistically significant value. *P* < 0.01.

^*d*^ The *P* values of 0.0428, 0.0110, and 0.0046 indicate statistically significant associations between *Prevotella* abundance and clinical stage, tumor size, and DSI, respectively.

### 16S rRNA sequencing reveals distinct features of the microbiome in cervical tissues

**Fig 1:**
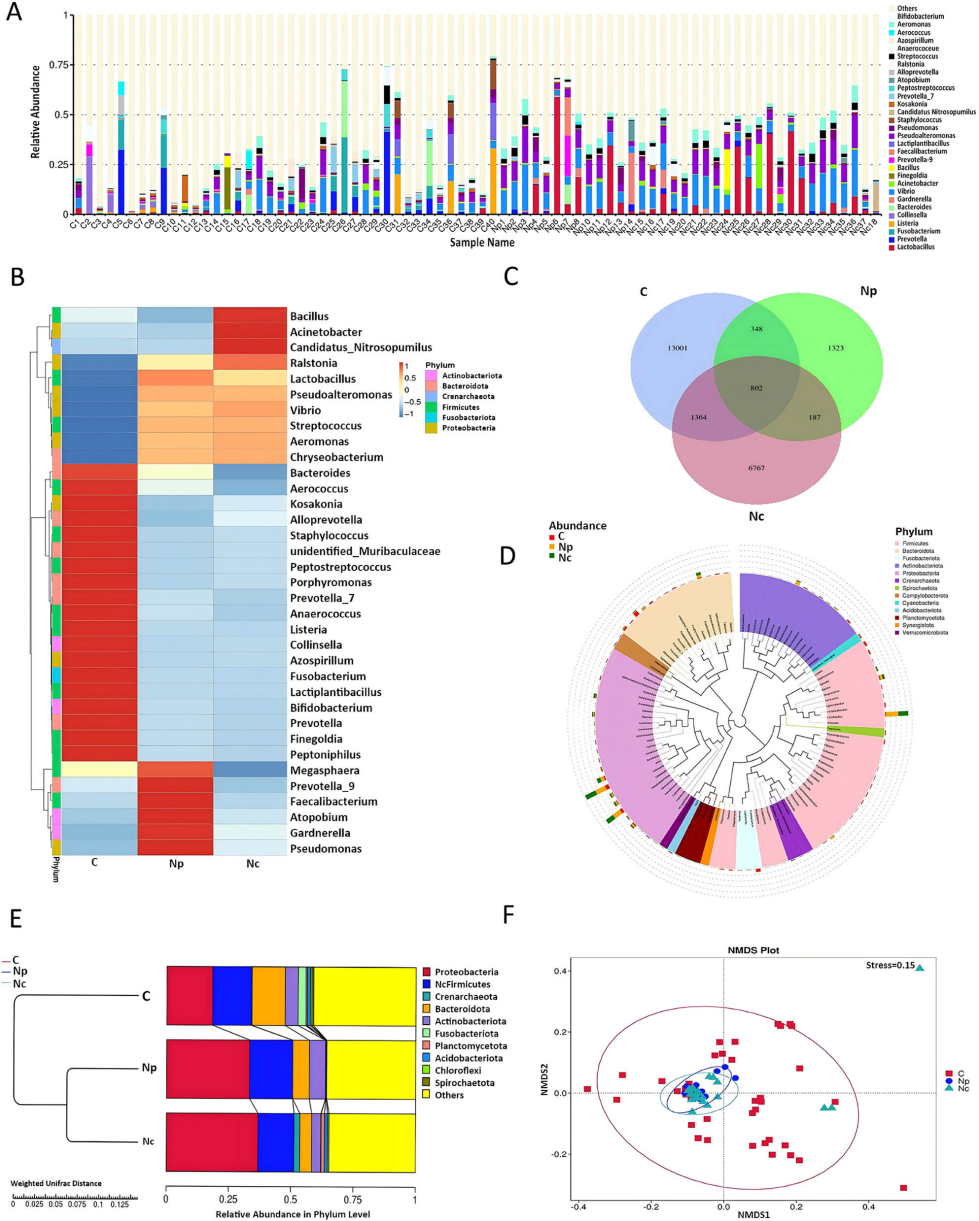
Analysis of the composition of the microbiota in cervical tissues. (**A**) Composition features of the microbiota in each group at the genus level. Different shades of color represent the different genera within a phylum. (**B**) Heatmap showing phylum-level species abundance in groups C, Np, and Nc. Rows are species, and columns are samples. The left dendrogram shows species clustering. Colors represent *Z*-score-normalized abundance values ranging from −1 (blue, low relative abundance) to 1 (red, high relative abundance). (**C**) Venn diagram of the signature sequences in the three groups. (**D**) Genus-level phylogenetic trees of the three groups. The colors of the branches and fan-shaped sections indicate their corresponding phyla, and the stacked bar diagram outside the fan ring indicates the abundance distribution information of the genus in different groups. (**E**) UPGMA analysis of the three groups using weighted UniFrac distance. (**F**) NMSD diagram illustrating the differences in the composition of the microbial communities among groups C, Np, and Nc according to the Jaccard distance. Each point represents a sample, and the distance between points reflects the differences in community structure based on the Bray–Curtis metric. C: cervical cancer tissues; Np: paracancerous tissues; Nc: normal cervical tissues; UPGMA: unweighted pair-group method with arithmetic mean.

By analyzing the clean 16S rRNA data after DADA2 denoising, we obtained a total of 24,021 ASVs, corresponding to 881 species, 1,374 genera, 568 families, 362 orders, 161 classes, 68 phyla, and 2 kingdoms (Table S2). The bacterial genera with high abundance included *Lactobacillus*, *Prevotella*, *Fusobacterium*, *Listeria*, *Collinsella*, and *Bacteroides* (Fig. 1A and B). Given the significant differences in the pathological features among cervical cancer tissues, normal cervical tissues, and paracancerous tissues, we explored the compositional diversity of the bacterial microbiome in the three groups. A Venn diagram of the microbial composition in the tissues revealed that the cervical cancer tissues had a greater variety of microorganisms on the basis of the ASVs (Fig. 1C). In addition, the genus-level evolutionary tree revealed a wider distribution of the microbiome in cervical cancer tissues than in the other groups (Fig. 1D).

The spectral diversity index revealed that although good coverage was detected, the alpha diversity of the bacterial microbiome differed little among the three groups (Fig. S1A through F). However, beta diversity, indicated by UPGMA and NMDS, significantly differed between cervical cancer tissues and the other two tissue types, and the Np and Nc samples appeared more similar (Fig. 1E and F). Additionally, these findings were verified by other beta diversity indices, such as distance matrix heatmaps and weighted UniFrac distances using Tukey’s test and the Kruskal–Wallis rank sum test (*P* < 0.05) (Fig. S1G through L).

These results revealed the overall patterns of microbial composition among the three groups and revealed that the microbial composition of cervical cancer tissues differed from that of normal cervical tissues and paracancerous tissues. Thus, we hypothesized that the intratumoral microbiome may change during the development of cervical cancer.

### Association of the relative abundance of the intratumoral microbiome with the clinical features and survival of patients with cervical cancer

**Fig 2:**
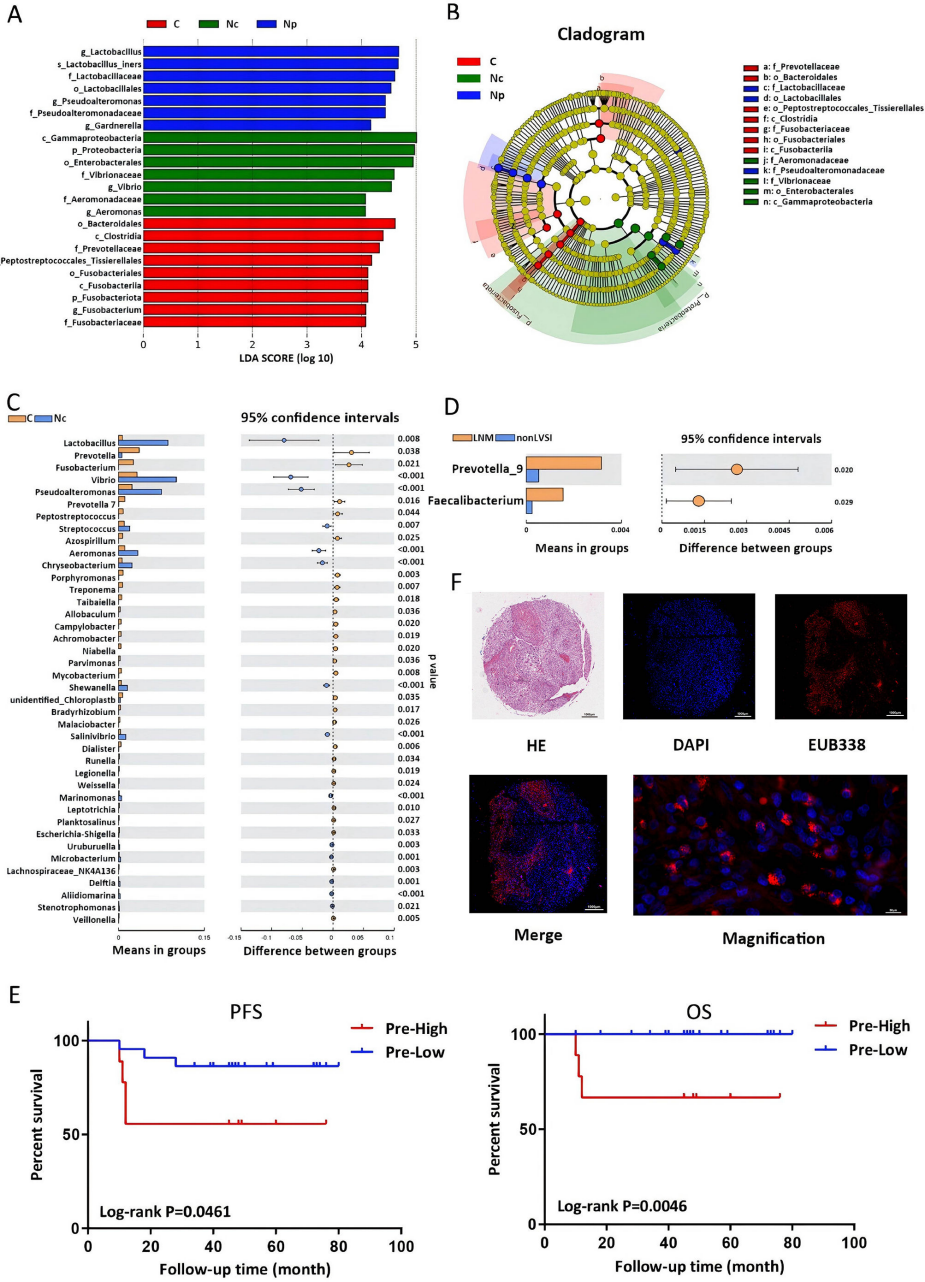
Species with significant variation in abundance between groups according to statistical tests. LEfSe analysis of the three groups: (**A**) Histogram of the LDA values quantified according to the proportions of different microbial species in the three groups of samples. (**B**) The phylogenetic tree shows the distribution of microbial differences in the three groups of samples. The size of the nodes represents the relative abundance of that node. (**C**) Statistical *t* test to identify species with significant differences between the three groups at each taxonomic level; (**D**) statistical *t* test between the LNM groups and the non-LVSI group. (**E**) Kaplan–Meier PFS and OS curves of cervical cancer patients in the high abundance *Prevotella* group compared with those in the low abundance *Prevotella* group, using the log-rank test. (**F**) Eub338 validation experiments show the presence of bacteria in the cervical TMA tissues. LEfSe: LDA effect size; LNM: lymph node metastasis; LVSI: lymphatic vascular space infiltration; PFS: progression-free survival; OS: overall survival; TMA: tissue microarray.

Next, a LEfSe histogram and evolutionary branching diagram were used to rank the microbes with the greatest differences in abundance between tumor tissues and non-tumor tissues (Fig. 2A and B). The LDA (threshold = 4) results revealed a greater abundance of *Lactobacillus* in paracancerous tissues and Vibrio in normal cervical tissues, whereas *Prevotella* and *Fusobacteriaceae* were more abundant in cancerous tissues. A *t* test analyzing the C and Nc groups revealed that *Lactobacillus* and *Prevotella* made the greatest contributions (Fig. 2C). Moreover, we evaluated the differences between lymph node metastasis (LNM) tissues and nonlymphatic vascular space infiltration (non-LVSI) tissues in the cervical cancer group (Fig. 2D) and reported that the abundances of *Prevotella*_9 and *Faecalibacterium* increased significantly in the LNM subgroup.

At the genus level, we analyzed the relationships between the relative abundance of *Prevotella/Lactobacillus* and the clinicopathological characteristics of cervical cancer patients. We found that the relative abundance of *Prevotella* was correlated with deep stromal invasion (DSI; *P* = 0.0046), tumor size (*P* = 0.011), and International Federation of Gynaecology and Obstetrics (FIGO) stage (*P* = 0.0428). However, there was no significant association between the relative abundance of *Lactobacillus* and these clinicopathological characteristics (Table 1). Progression-free survival (PFS) and overall survival (OS) were lower in cervical cancer patients with a high abundance of *Prevotella* than in those with a low *Prevotella* abundance (Fig. 2E, log-rank *P* = 0.0461/0.0046). However, no significant association was found between *Lactobacillus* abundance and PFS or OS (Fig. S2). Limitations in sample size and the heterogeneity of clinical data reduce the sensitivity of statistical analyses, potentially masking subtle but genuine associations. Finally, we performed fluorescence *in situ* hybridization staining with the EUB338 probe to determine the location of the microbiome. The results revealed that the microbiome was located primarily in the cytoplasm of cervical cancer cells and was partly distributed within the tissue stroma (Fig. 2F).

### Function of *Prevotella bivia* and *Lactobacillus iners* in cervical cancer *in vitro*

Considering that bacterial supernatants contain many molecules and metabolites, *in vivo* and *in vitro* experiments were designed to study the functions of *Lactobacillus* and *Prevotella* supernatants. We specifically chose *Prevotella bivia* and *Lactobacillus iners* to provide the supernatant because the relative abundances of these two species are relatively high in cervical tissues. The human cervical cancer cell lines SiHa and HeLa were also utilized in our study.

**Fig 3:**
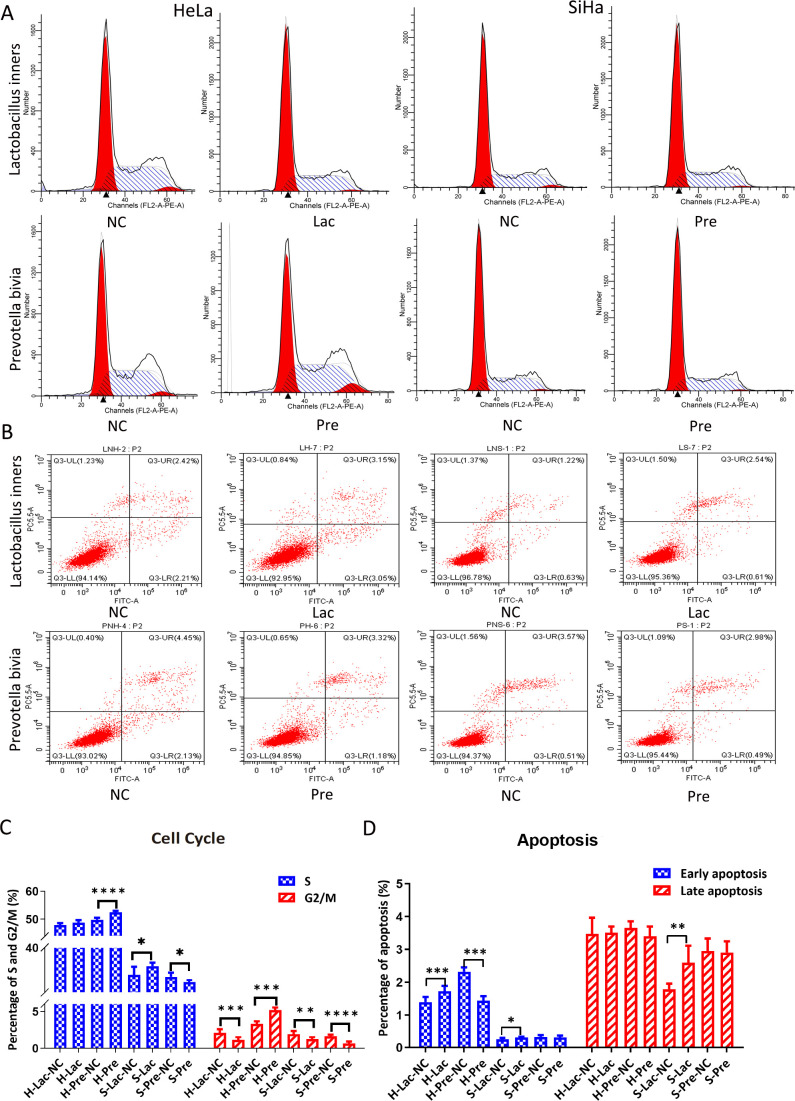
Effects of *Lactobacillus iners*/*Prevotella bivia* supernatant on cell cycle distribution and apoptosis. (**A and B**) Cell cycle distributions and apoptosis of HeLa and SiHa cells after the 48 h co-culture with the supernatant by flow cytometry. (**C**) Cell cycle S/G2/M phase distribution was expressed as the percentage of HeLa and SiHa cells after the 48 h co-culture with the supernatant. (**D**) The quantification of the early and late apoptosis in tumor cells as shown in panel B. *P < 0.05, **P < 0.01, ***P < 0.001, compared with the control groups.

The effects of *L. iners*/*P. bivia* on cervical cancer cell viability and apoptosis were determined by flow cytometry. Compared with those in the control groups, SiHa and HeLa cells cocultured with the *L. iners* supernatant arrested in the S, G2/M phase, and HeLa cells cocultured with the *P. bivia* supernatant were more likely to be accelerated in the S, G2/M phase (Fig. 3A and C). The *L. iners* supernatant led to an increase in the numbers of early and late apoptotic SiHa cells and the number of early apoptotic HeLa cells, whereas the *P. bivia* supernatant led to a decrease in the number of early apoptotic cells for both cell types (Fig. 3B and D).

**Fig 4:**
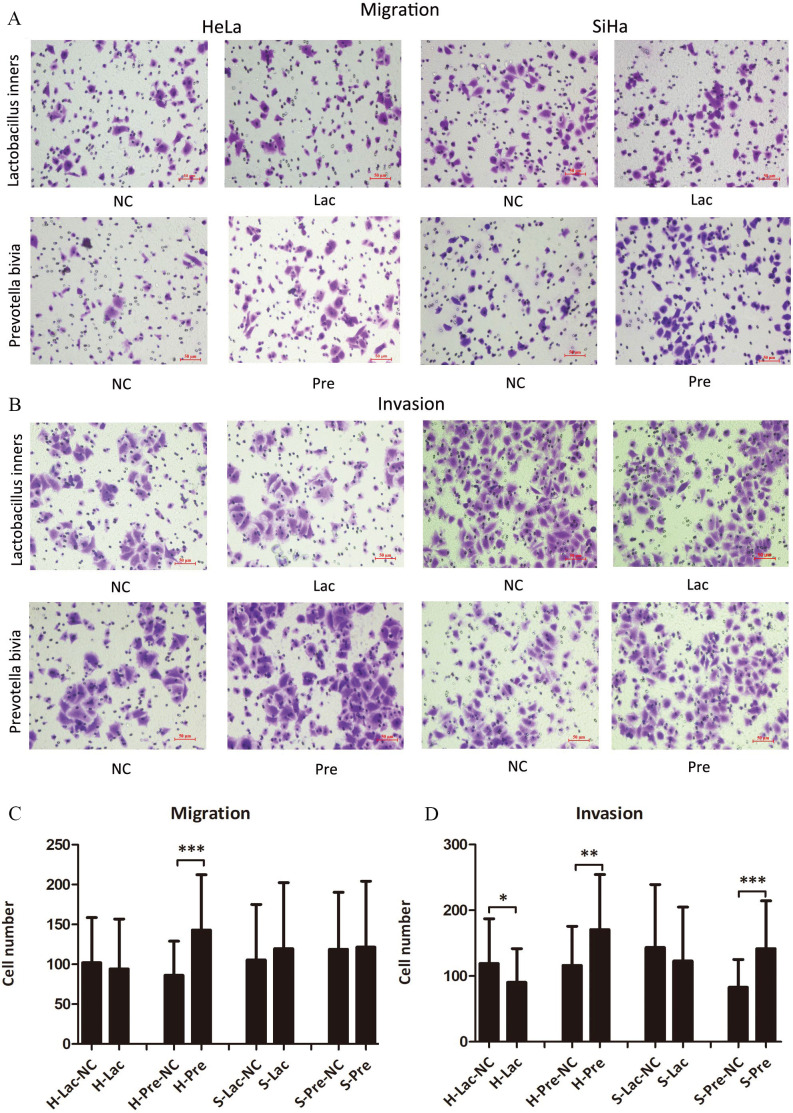
Effects of *Lactobacillus iners*/*Prevotella bivia* supernatant on cell migration and invasion. (**A**) Transwell experiments to verify the migration ability of the HeLa/SiHa cervical cancer cell line after treatment with *L. iners*/*P. bivia* supernatant (*n* = 3). (**B**) Transwell experiments were performed to verify the invasiveness of the HeLa/SiHa cervical cancer cell lines after treatment with *L. iners*/*P. bivia* supernatant (*n* = 3). (**C**) As shown in panel A, quantification of migrated cervical cancer cell line cells after supernatant treatment compared to the control group (**D**). As shown in panel B, quantification of invaded cervical cancer cell line cells after supernatant treatment compared to the control group; *P < 0.05, **P < 0.01, ***P < 0.001, compared with the control groups.

A transwell assay was used to detect the effects of *L. iners*/*P. bivia* on cervical cancer cells. Compared with those in the control groups, the number of migrated and invasive cells increased significantly in the HeLa *P. bivia* supernatant group, and the number of invasive cells also increased markedly in the SiHa *P. bivia* supernatant group. In addition, *L. iners* supernatant inhibited the migration of SiHa cells (Fig. 4A through D).

### Function of the *L. iners*/*P. bivia in vivo*

**Fig 5:**
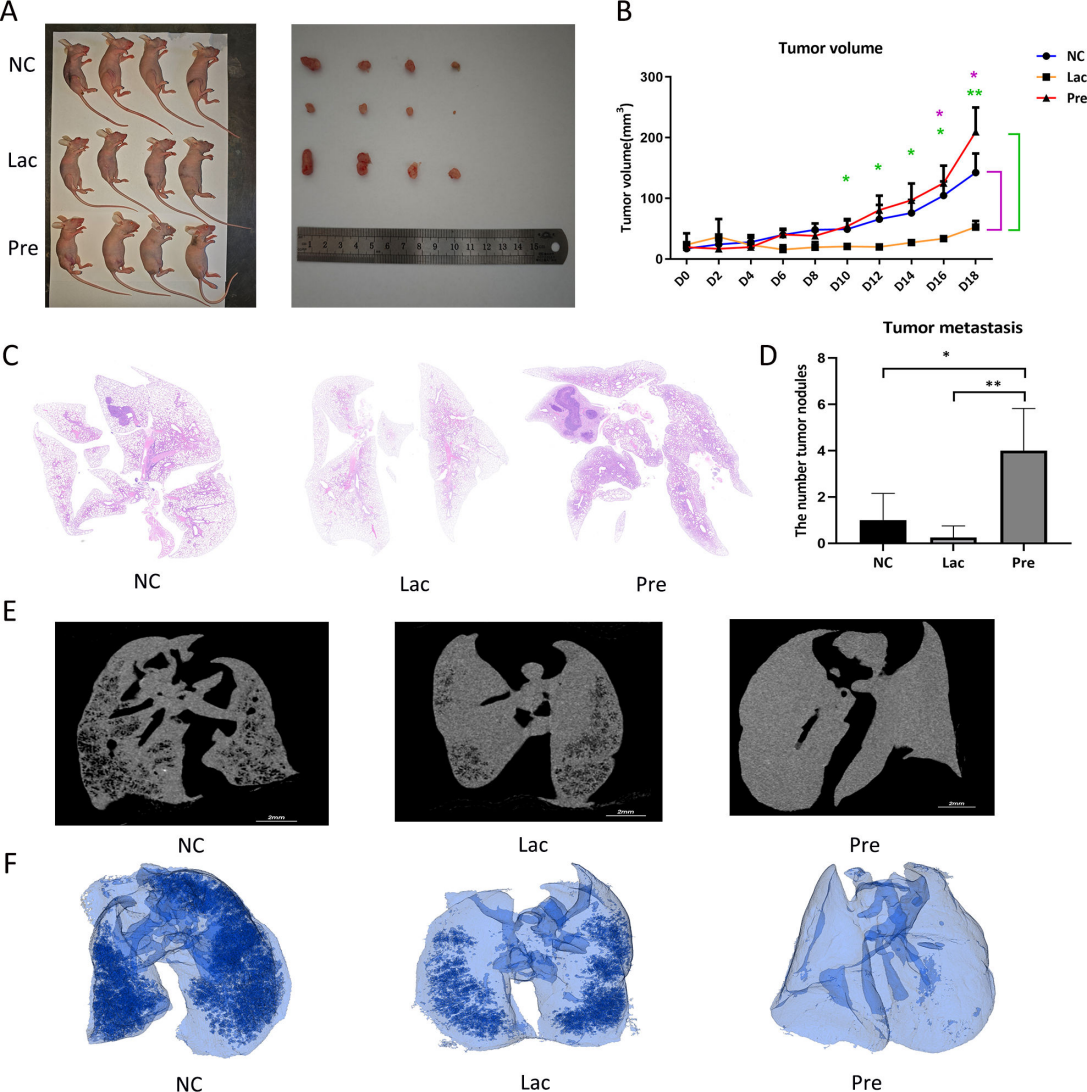
Effects of *Lactobacillus iners*/*Prevotella bivia* supernatant on tumor growth and metastasis *in vivo*. (**A**) HeLa cells were subcutaneously injected into nude mice. The supernatant or PBS was administered intraperitoneally every other day. The tumor volumes were measured continuously for 18 days, after which the mice were euthanized, and the tumors were excised. (**B**) Quantification of subcutaneous tumor volumes in the nude mice described in panel **A**. (**C**) HeLa cells were injected via the tail vein. The supernatant and PBS were administered as described in panel **A**. After 30 days, the mice were sacrificed, and the lung tissues were subjected to HE staining to visualize the metastatic tumor nodules. (**D**) Quantitative analysis of the pulmonary metastatic nodules shown in panel **C**. (**E**) Representative lung CT scans of the three experimental groups. (**F**) 3D reconstructed images of lungs from the three groups. The data are presented as the mean ± SEM. *P < 0.05, **P < 0.01.

The use of a xenograft model can effectively reduce the interference caused by the complexity of the host immune system, allowing us to more clearly observe the effects of bacterial metabolites on tumor cells. Therefore, in this study, a xenograft model was selected for *in vivo* experimental validation. To determine whether *L. iners*/*P. bivia* could influence tumor growth *in vivo*, HeLa cells were injected subcutaneously into athymic nude mice. Afterward, the supernatants of the two species and PBS were injected into the abdominal cavities of the mice every 2 days, after which the tumor volume was measured for 18 days (Fig. 5A and B). As shown in Fig. 5B, the tumors in the *L. iners* group grew at a slower rate than the tumors in the *P. bivia and* control groups did after day 10 and day 16. Additionally, we constructed a lung metastasis model in nude mice by injecting HeLa cells via the tail vein, after which the supernatants of these two species and PBS were injected into the abdominal cavity every 2 days. After 1 month, the number of tumor nodules in the lungs of the *P. bivia* group was greater than that in the *L. iners* and control groups (Fig. 5C and D and Fig. S3A). Interestingly, less residual gas in the lungs was detected in the *L. iners*/*P. bivia* groups than in the control group, and the difference in the *P. bivia* group was more apparent (Fig. 5E and F and Fig. S3B and C). These *in vivo* and *in vitro* data suggest that *L. iners* might inhibit tumor cell proliferation, whereas *P. bivia* may promote cervical cancer metastasis.

### Possible mechanisms of *L. iners/P. bivia* in cervical cancer

**Fig 6:**
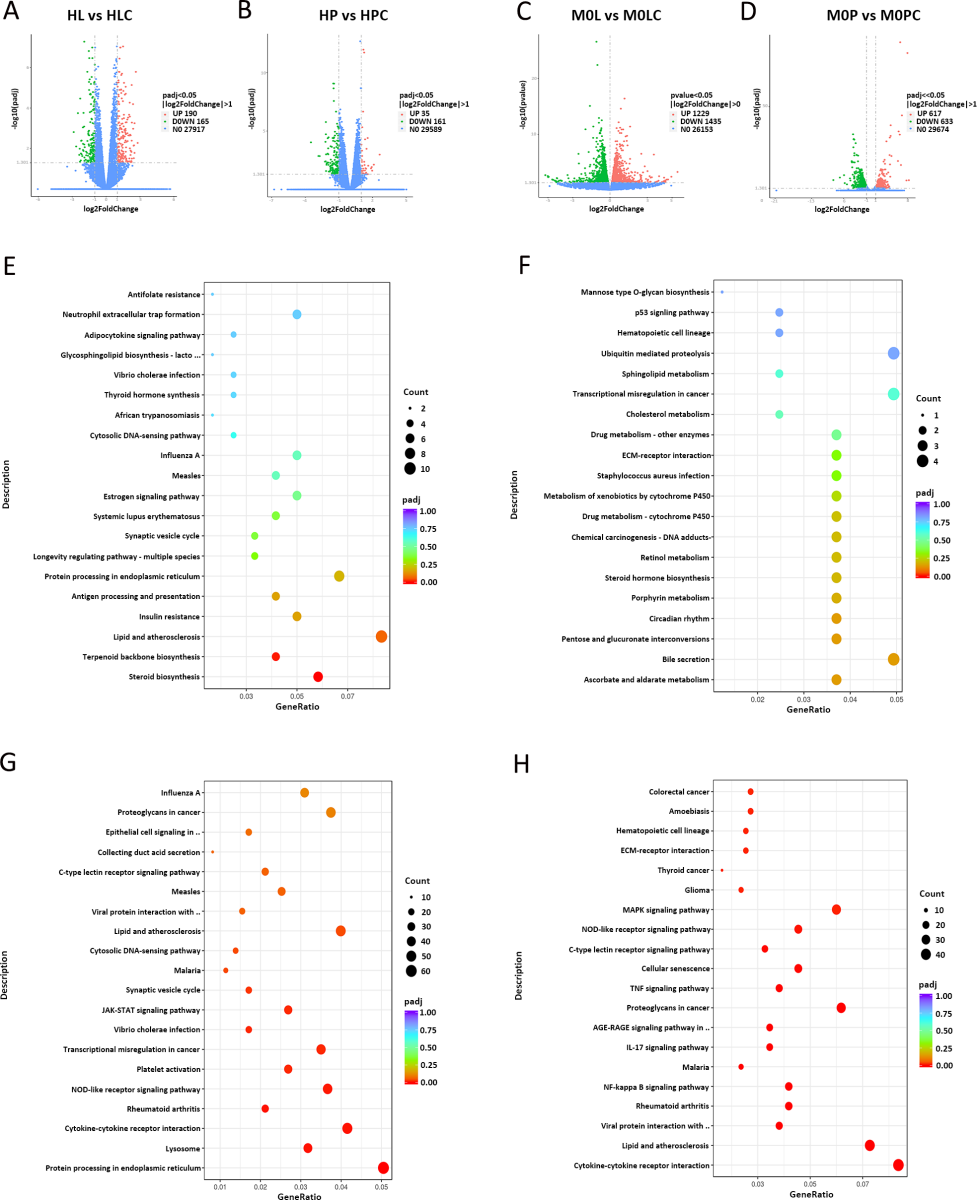
Transcriptome sequencing of tumor cells and macrophages. (**A–D**) Volcano plots of the enrichment of all DEGs in HeLa cells and macrophages after coculture with *Lactobacillus iners* and *Prevotella bivia* supernatant for 48 h. (**E and F**) Scatter plot of KEGG pathway enrichment. Each circle represents a KEGG pathway. Thus, the greater the enrichment factor is, the more significant the enrichment level of DEGs in the pathway. KEGG, Kyoto Encyclopedia of Genes and Genomes. DEG, differentially expressed gene.

**Fig 7:**
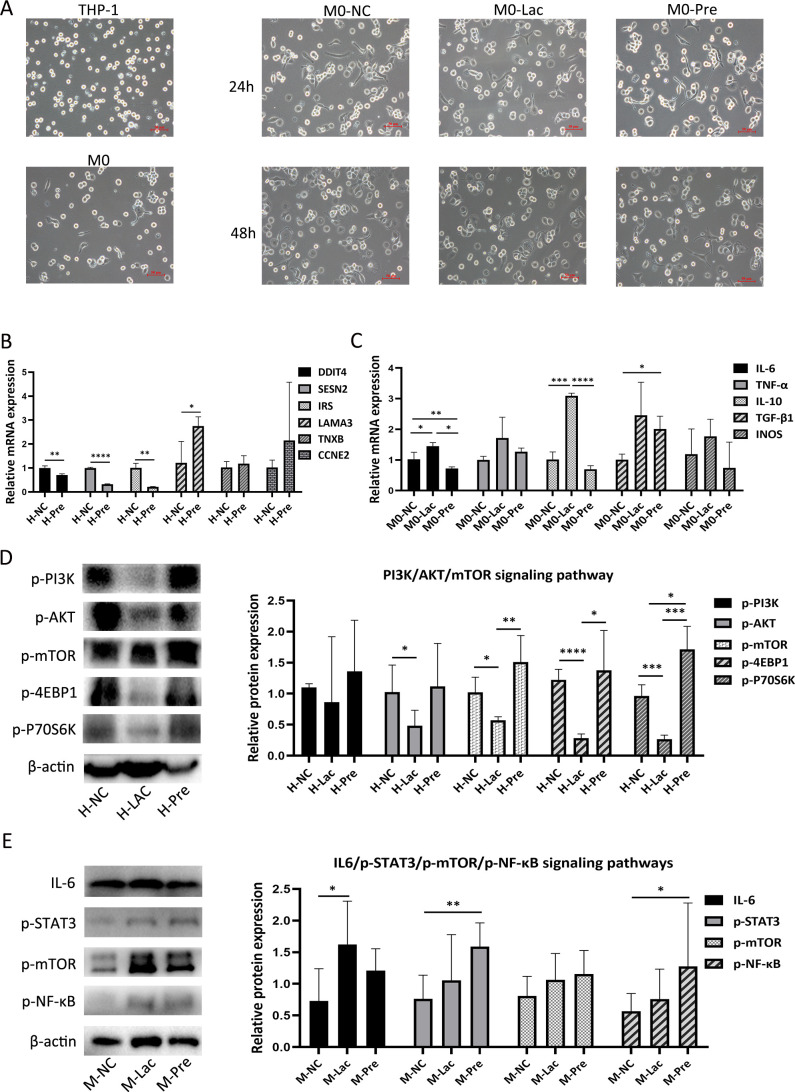
Morphological changes in macrophages and the verification of the transcriptome sequencing signaling pathways. (**A**) After 24 hours of the PMA induction, THP-1 cells were stimulated to become M0 cells, and then, the M0 cells were cocultured with *Lactobacillus iners* and *Prevotella bivia* supernatants for 24 and 48 hours to observe the morphological changes. (**B**) PCR verification of the downstream molecules of the PI3K/AKT/mTOR pathway in HeLa cells. (**C**) PCR verification of the M1/M2 markers in the macrophages. (**D**) Effects of *Lactobacillus iners* and *Prevotella bivia* supernatants on the levels of phosphorylated PI3K/AKT/mTOR signaling pathway, with its quantification in tumor cells. (**E**) Effects of *Lactobacillus iners* and *Prevotella bivia* supernatants on signaling pathways associated with macrophage polarization. PMA, phorbol 12-myristate 13-acetate. PCR, polymerase chain reaction. *P < 0.05, **P < 0.01, ***P < 0.001, ****P < 0.001, compared with the control groups.

To elucidate the potential regulatory networks of the intratumoral microbiome in cervical cancer, we performed transcriptome sequencing of HeLa cells and macrophages after coculture with *L. iners*/*P. bivia* supernatant. The analysis revealed that *L. iners* resulted in 190 upregulated messenger RNAs (mRNAs) and 165 downregulated mRNAs in cervical cancer cells, whereas *P. bivia* led to 35 upregulated and 161 downregulated mRNAs (Fig. 6A and B; Tables S3 and S4). In macrophages, *L. iners* resulted in 1,229 upregulated and 547 downregulated mRNAs, and *P. bivia* led to 617 upregulated and 633 downregulated mRNAs (Fig. 6C and D; Tables S5 and S6). Overall, *L. iners* caused more mRNAs to be differentially expressed in these two cell lines, and macrophages were more susceptible to regulation by both bacterial supernatants. In addition, we observed morphological changes in macrophages after coculture with *L. iners*/*P. bivia* supernatant. Compared with those in the control group, the macrophages in both the *P. bivia* group and the *L. iners* group became slenderer after 24 h of coculture (Fig. 7A).

KEGG pathway enrichment analyses were subsequently performed on all the differentially expressed mRNAs to identify the possible signaling pathways involved. The results revealed that the genes in cancer cells regulated by *L. iners* were associated primarily with functions related to increased steroid biosynthesis and terpenoid backbone biosynthesis, decreased insulin resistance, and the PI3K–Akt signaling pathway (Fig. 6E; Table S7). The genes in cancer cells regulated by *P. bivia* were associated mainly with increased oxidative phosphorylation and human papillomavirus (HPV) infection, decreased ascorbate and aldarate metabolism, and other metabolic signaling pathways (Fig. 6F; Table S8). Additionally, the expression of genes related to the PI3K–Akt signaling pathway, such as DDIT4, SESN2, IRS, and LAMA3, determined by PCR was consistent with that determined by RNA sequencing (Fig. 7B; Table S1). We then detected key proteins in the PI3K–AKT pathway via western blotting and found that *L. iners* significantly decreased the levels of phosphorylated AKT/mTOR/4EBP1/P70S6K, whereas *P. bivia* significantly increased the levels of phosphorylated mTOR/4EBP1/P70S6K (Fig. 7D). Moreover, the macrophage genes regulated by *L. iners* were associated mainly with activation of the synaptic vesicle cycle, decreased protein processing in the endoplasmic reticulum, and suppressed JAK-STAT signaling (Fig. 6G; Table S9). The genes in macrophages regulated by *P. bivia* were associated mainly with increased cytokine–cytokine receptor interactions, activation of the NF-κB and JAK-STAT signaling pathways, and suppression of other signaling pathways, including proteoglycans in cancer, protein digestion, and absorption (Fig. 6H; Table S10). Next, to investigate the effects of *L. iners*/*P. bivia* on M1/M2 macrophage polarization, we detected the molecular markers of M1 (IL-6, INOS, and TNF-α) and M2 (IL-10 and TGF-β1) macrophages via PCR. *P. bivia* increased the mRNA expression of TGF-β1 and decreased that of IL-6. However, the effect of *L. iners* on macrophage polarization is not clear, as this supernatant increased the mRNA expression of both IL-6 and IL-10 (Fig. 7C). Furthermore, western blotting was carried out to examine the specific molecular pathways modulated by *P. bivia* that led to M2 macrophage polarization, and the results revealed that phosphorylated STAT3 and NF-κB expression increased significantly (Fig. 7E).

### Correlation analysis of the intratumoral microbiome and the metabolome of cervical cancer cells

**Fig 8:**
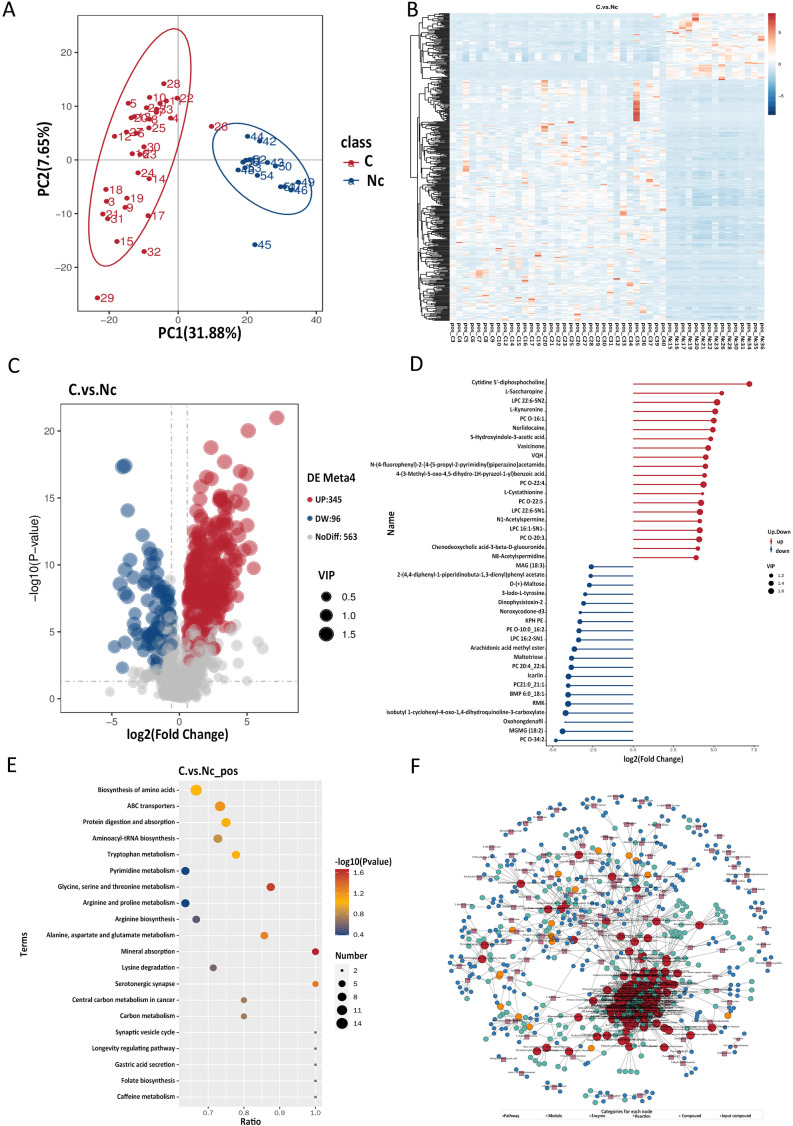
Metabolomic sequencing of cervical cancer tissue and normal cervical tissue. (**A**) PCA of the metabolites of the C and Nc groups. (**B**) Metabolite heatmap of the two groups. (**C**) Volcano plots of the enrichment of all the metabolites in the C and Nc groups. (**D**) Metabolite matchstick diagram analysis between the C and Nc groups. The blue dots represent decreased enrichment, and the red dots represent increased enrichment. The length of the rod represents the magnitude of log2(fold change), and the size of the dot represents the size of the VIP value. (**E**) Scatter plot of KEGG pathway enrichment. (**F**) KEGG network regulation diagram. The red dots represent metabolic pathways, the yellow dots represent the relevant regulatory enzyme information of a substance, the green dots represent the background material of a metabolic pathway, the purple dots represent the molecular module information of a class of substances, the blue dots represent the chemical interactions of a substance, and the green squares represent the difference observed in this comparison. PCA, principal component analysis. VIP, variable importance in the projection.

Nontargeted metabolomic sequencing revealed differences between the C and Nc groups (Fig. 8A through D; Table S11). KEGG analysis of these differentially abundant metabolites revealed significant changes in mineral absorption, glycine/serine and threonine metabolism, serotonergic synapses, alanine/aspartate metabolism, and glutamate metabolism (Fig. 8E; Table S12). The KEGG regulatory network diffusion algorithm revealed the highly clustered and complex interactions among metabolic pathways and potential target enzymes and metabolites, highlighting the importance of metabolism in cervical cancer (Fig. 8F). Comparative metabolite analysis of the tumor tissues with high and low *L. iners*/*P. bivia* abundance revealed their apparent differences (Fig. S4). KEGG classification revealed that the differentially abundant metabolites were associated mainly with amino acid metabolism, carbohydrate metabolism, and lipid metabolism (Fig. S5). Furthermore, metabolomic analysis of the *L. iners*/*P. bivia* supernatants revealed similar alterations in the KEGG classifications (Fig. S6), but the specific differentially abundant metabolites, such as butenoyl-PAF in the *L. iners* group and glycine in the *P. bivia* group, were inconsistent with those in the tumor tissue (Fig. S7).

## DISCUSSION

An increasing number of studies have suggested the presence of bacterial communities within different types of solid tumors, including cervical cancer tumors. However, the precise roles these communities play in tumor progression remain to be elucidated. Huang et al. reported that high intratumoral levels of *Fusobacterium nucleatum* were correlated with tumor differentiation, poor survival outcomes of cervical cancer patients, and cancer stem cell (CSC) characteristics in patients with cervical cancer. However, only the presence of *F. nucleatum* was verified, and no mechanistic experiments were conducted (18). To comprehensively characterize the microbial landscape of cervical tissues during the progression from normal tissue to malignant tissue, we performed 16S RNA sequencing and follow-up experiments.

Unlike that observed with hepatocellular carcinoma (19), the intratumoral microbial heterogeneity of cervical cancer tissues is greater than that of non-tumor tissues, which is reflected mainly in the increased beta diversity, which is consistent with changes in the vaginal flora (20, 21). Moreover, these similar microbial changes may also explain the origin of the intratumoral bacteria. The vagina is an open lacunar organ, and bacteria may easily penetrate the mucosa and enter the tissues, including tumor tissues. In this study, we selected two bacteria (*L. iners* and *P. bivia*) on the basis of the relationships between their differential abundance and clinicopathological factors.

Live bacteria may contaminate cell media and affect cell growth; therefore, we used the bacterial culture supernatant, which has been commonly used in other studies (22–24). In addition to researching the influence of intratumoral bacteria on the biological behavior of tumor cells, we also investigated the influence of intratumoral bacteria on macrophage polarization. At present, it is generally believed that the tumor immune microenvironment plays important roles in the occurrence and development of cervical cancers (25, 26), and the polarization of tumor-related macrophages toward the M2 phenotype, which are the most abundant and play key roles in cervical tumor immune escape in cervical tumors (27–31).

As shown in the Results section, overall, *L. iners* and *P. bivia* had opposite effects on the biological behavior of tumor cells. Specifically, *L. iners* inhibited the proliferation of tumor cells but promoted their apoptosis, whereas *P. bivia* significantly promoted the migration and invasion of cervical tumor cells. Furthermore, the data from the *in vivo* animal tumor models and *in vitro* cell experiments were consistent. In addition, transcriptome sequencing verified the effects of these two strains on the phosphorylated PI3K/AKT/mTOR signaling pathway, which has been frequently confirmed as a carcinogenic pathway and therapeutic target for cervical cancer. Moreover, infection with high-risk HPV can promote activation of the PI3K/AKT/mTOR pathway (32–36). Therefore, we believe that an imbalance in the intratumoral flora and infection with high-risk HPV may synergistically affect the occurrence and development of cervical cancer. Activation of the PI3K/Akt/mTOR signaling pathway induces the phosphorylation of the mTOR complex 1 substrates 4E-BP1 and S6K, which induces the functional protein translational machinery and inhibits autophagy during early virus–host cell interactions (32). Our experimental results revealed that corresponding changes in 4E-BP1 and S6K expression occurred. With respect to the morphological changes and cell surface marker expression of macrophages and alterations in polarization-related pathways, we believe that *P. bivia* promotes M2 macrophage polarization, but the role of *L. iners* is unclear. A previous study demonstrated that M2 macrophages are significantly elongated compared with M1 and unstimulated M0 macrophages (37). Phosphorylated STAT3 and NF-κB have also been shown to promote M2 macrophage polarization (38–42).

Numerous studies have shown that the tumor microflora is closely related to the tumor cell metabolome (8, 43–46). Our analyses focused on three main areas: the overall relationship between the tumor metabolome and the intratumoral microbiome, including comparisons of the C and Nc groups, and the differences in the metabolomes of tumor tissues with high abundances of *L. iners*/*P. bivia* and tumor tissues with low abundances of these bacteria; and the different effects of the *L. iners*/*P. bivia* supernatants and control treatment. Considering the metabolic differences between cervical tumor tissue and normal tissue determined by KEGG analysis, we considered that the differentially abundant metabolites and their associated networks may be involved in the development of cervical cancer. Because tissue metabolites are derived from a wide range of sources, including tumor cells, mesenchymal cells, and intratumoral bacteria, the changes in metabolite abundance in the supernatant may not match those in the tissue. However, their pathway classifications, such as amino acid metabolism, were similar, which is consistent with data from other tumor types, such as intrahepatic cholangiocarcinoma (8, 47). Focusing on the differentially abundant metabolites in the supernatant group, we found that the level of glycine was significantly increased in the *P. bivia* group, which has been reported to be detrimental to proliferating cancer cells (48, 49), and a high glycine concentration in the tumor microenvironment can be considered a clinical indicator of poor prognosis (50). Moreover, glycine affects macrophage polarization via cell signaling pathways (e.g., NF-κB, NRF2, and Akt) and microRNAs (51). Future work will focus on exploring the specific functions of these metabolites.

In conclusion, the microbiome in cervical cancer tissues significantly differed from that in normal cervical tissues and was significantly correlated with clinicopathological features and survival prognosis. Moreover, the tumor microbiome affects the biological behavior of cervical tumor cells and the polarization of macrophages through metabolite production, thus playing important roles in the occurrence and development of cervical cancer.

### Limitations of the study

This study has several limitations. First, we focused on only two representative bacterial species, *L. iners*/*P. bivia*, which limits the generalizability of our findings. Further research is needed to explore the functions and mechanisms of other bacterial taxa within the cervical tumor microbiome. Second, our functional experiments utilized bacterial culture supernatants containing mixed metabolites and components. This approach restricts the identification of specific active factors responsible for the observed effects. Additionally, supernatants cannot replicate direct physical interactions between live bacteria and tumor cells, which may be critical in tumor progression. Future studies should incorporate live bacterial coculture systems and *in vivo* models to clarify these interactions and their impact on tumor biology. Overall, while this study lays important groundwork for understanding key bacteria in cervical cancer, more comprehensive and mechanistic investigations are necessary to fully elucidate the roles of the intratumoral microbiome in cervical carcinogenesis and to inform potential therapeutic strategies.

## Data Availability

The raw 16S rDNA sequencing, transcriptome sequencing, and the nontargeted metabolomic sequencing reads were all deposited into the Science Data Bank (https://www.scidb.cn/anonymous/eVF6NjNp). All the data are available upon request from the authors.

## SUPPLEMENTAL MATERIAL

Fig. S1 mbio.00374-26-s0001.tif Analysis data from multi-omics sequencing.

Fig. S2 mbio.00374-26-s0002.tif PFS and OS in Lac-High and Lac-Low group.

Fig. S3 mbio.00374-26-s0003.tif HE staining, lung CT scans, and 3D images of the lungs from each group.

Fig. S4 mbio.00374-26-s0004.tif Analysis data from multi-omics sequencing.

Fig. S5 mbio.00374-26-s0005.tif Analysis data from multi-omics sequencing.

Fig. S6 mbio.00374-26-s0006.tif Analysis data from multi-omics sequencing.

Fig. S7 mbio.00374-26-s0007.tif Analysis data from multi-omics sequencing.

Legends mbio.00374-26-s0008.docx Legends for supplemental items.

Tables S1 and S2 mbio.00374-26-s0009.xlsx qPCR primers and 16S rRNA Data.

Tables S3-S15 mbio.00374-26-s0010.xlsx Analysis data from multi-omics sequencing, software packages and algorithms, and clinical variables and lifestyle factors.

ASM does not own the copyrights to Supplemental Material that may be linked to, or accessed through, an article. The authors have granted ASM a non-exclusive, world-wide license to publish the Supplemental Material files. Please contact the corresponding author directly for reuse.
