# Supplementary figures and images for: Prevotella bivia promotes cervical cancer progression and modulates macrophage polarization, while Lactobacillus iners suppresses these processes: evidence from multiomics analysis
Source: mBio. 2026 Jun 15;17(7):e00374-26. doi: 10.1128/mbio.00374-26 (PMC13344011; doi:10.1128/mbio.00374-26)

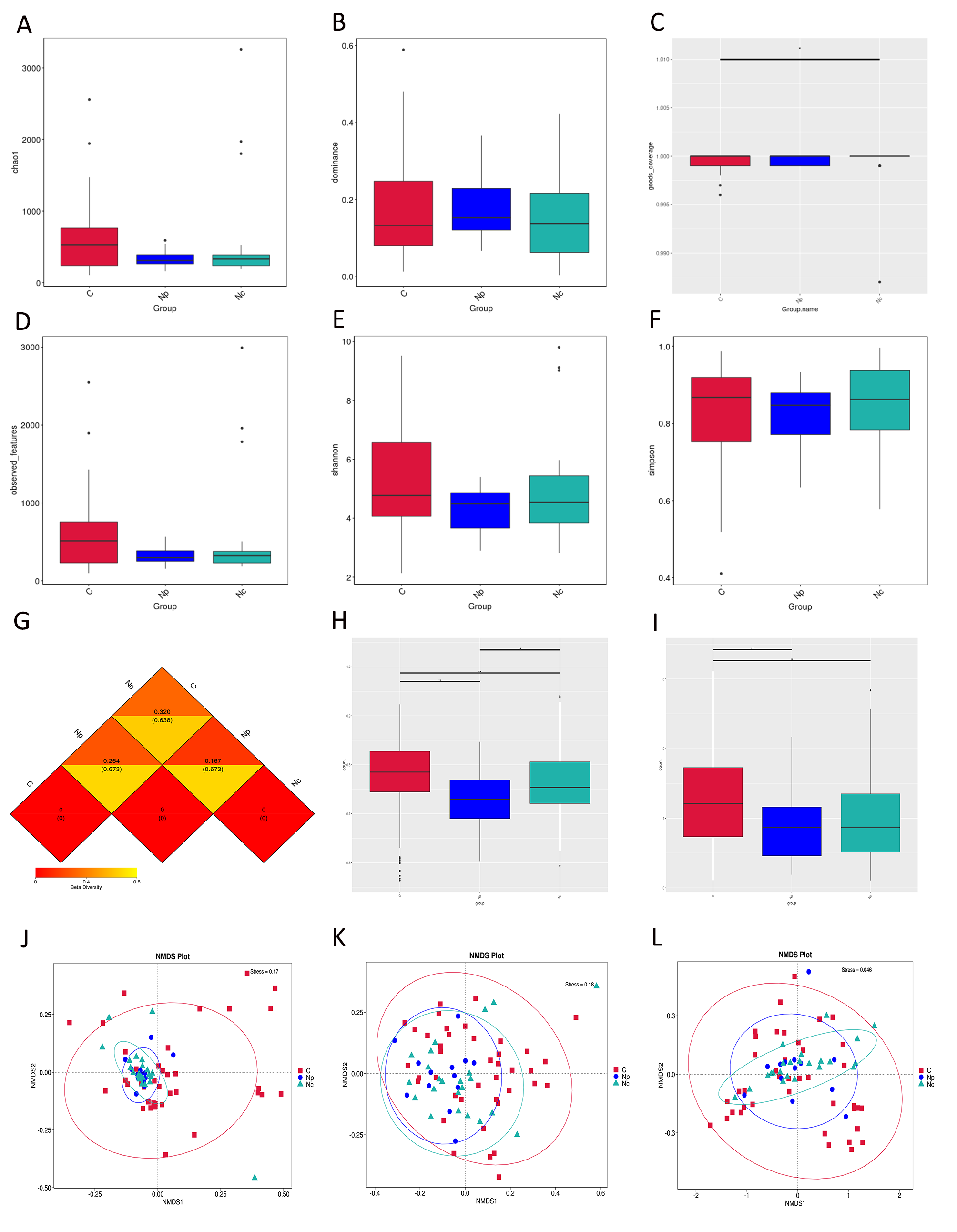

Supplement: Fig. S1 — Analysis data from multi-omics sequencing. [file mbio.00374-26-s0001.tif]

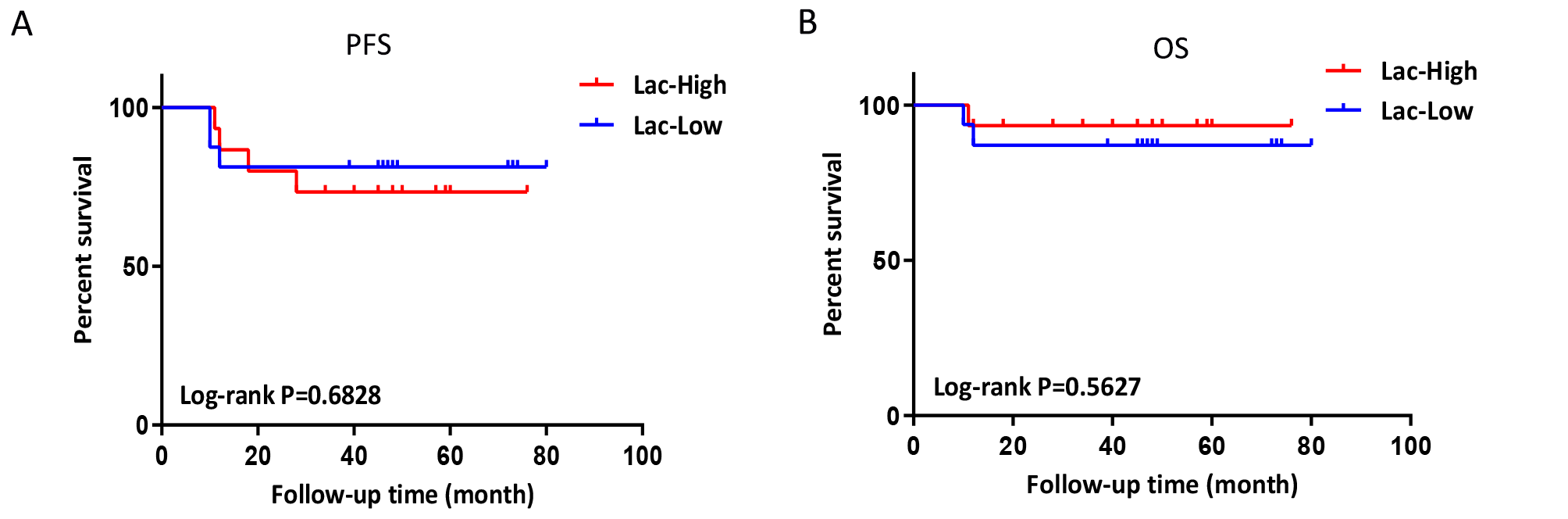

Supplement: Fig. S2 — PFS and OS in Lac-High and Lac-Low group. [file mbio.00374-26-s0002.tif]

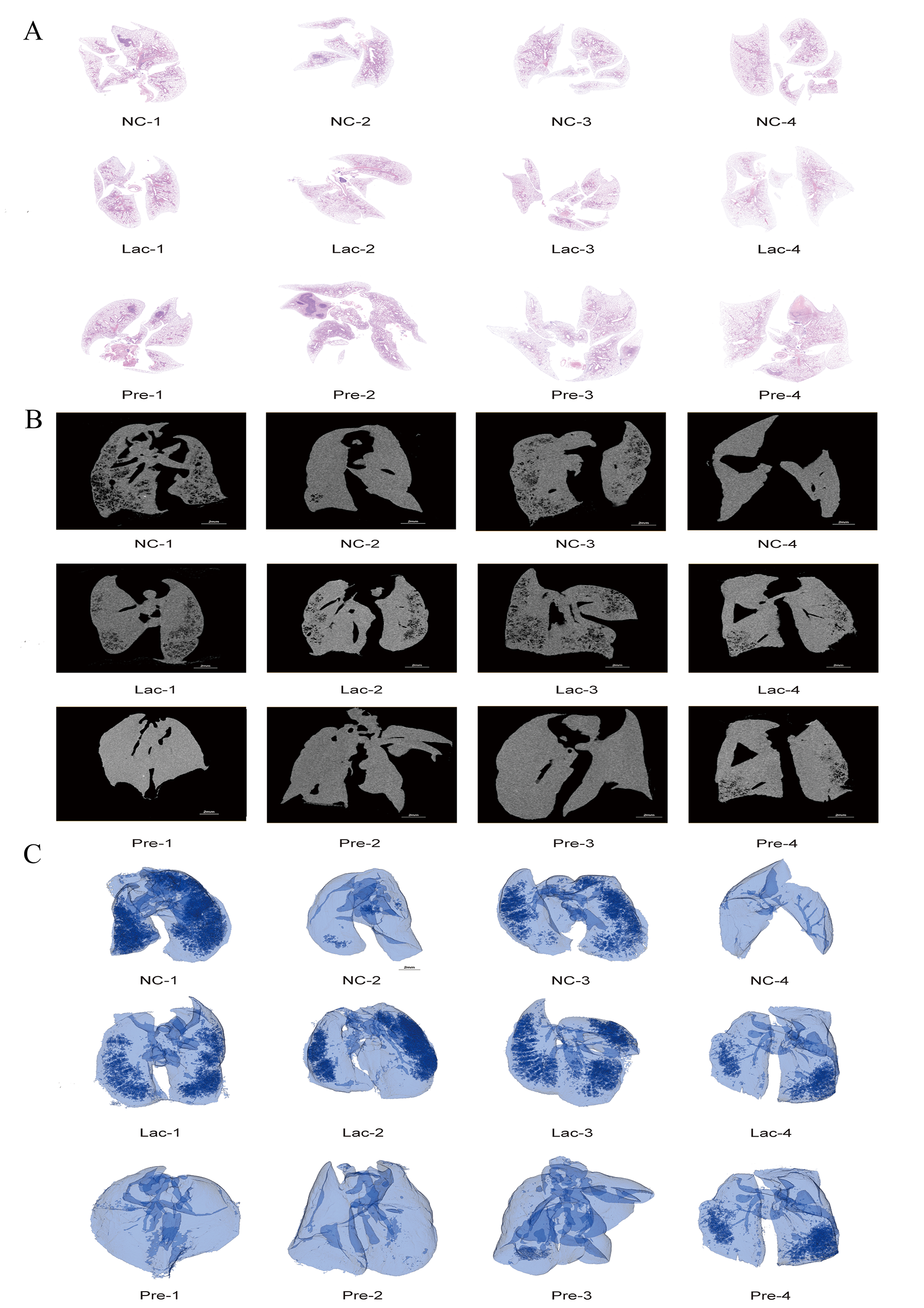

Supplement: Fig. S3 — HE staining, lung CT scans, and 3D images of the lungs from each group. [file mbio.00374-26-s0003.tif]

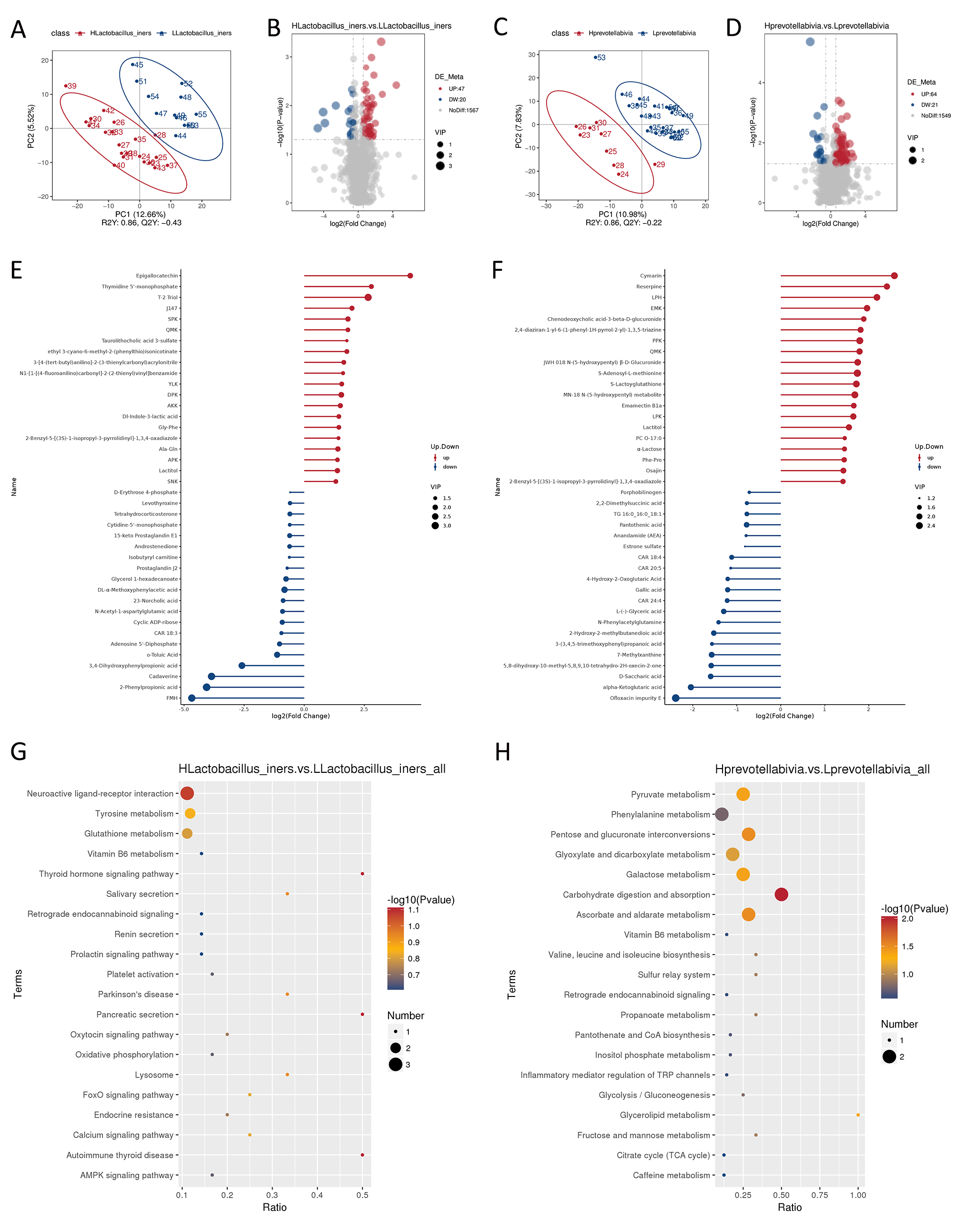

Supplement: Fig. S4 — Analysis data from multi-omics sequencing. [file mbio.00374-26-s0004.tif]

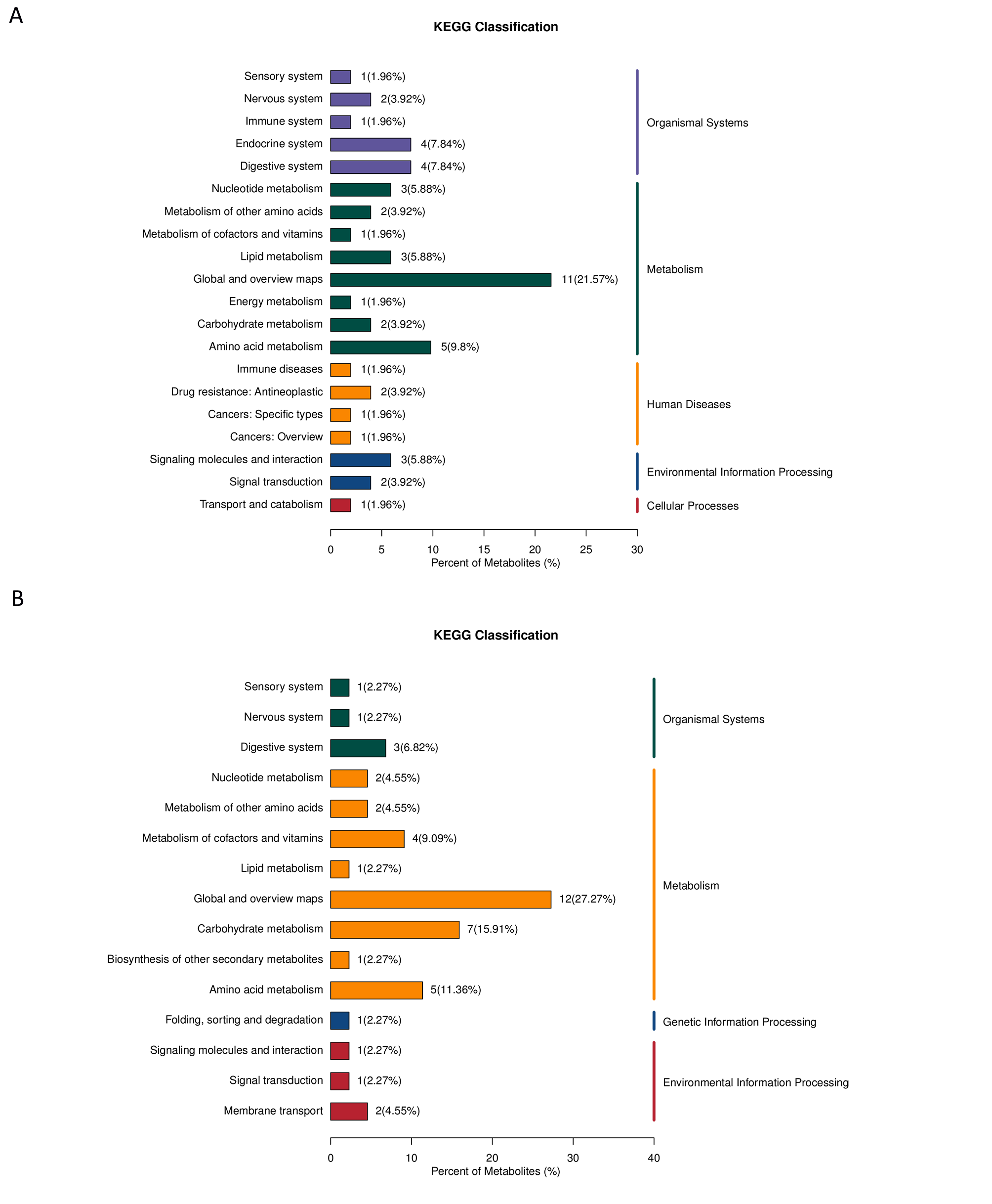

Supplement: Fig. S5 — Analysis data from multi-omics sequencing. [file mbio.00374-26-s0005.tif]

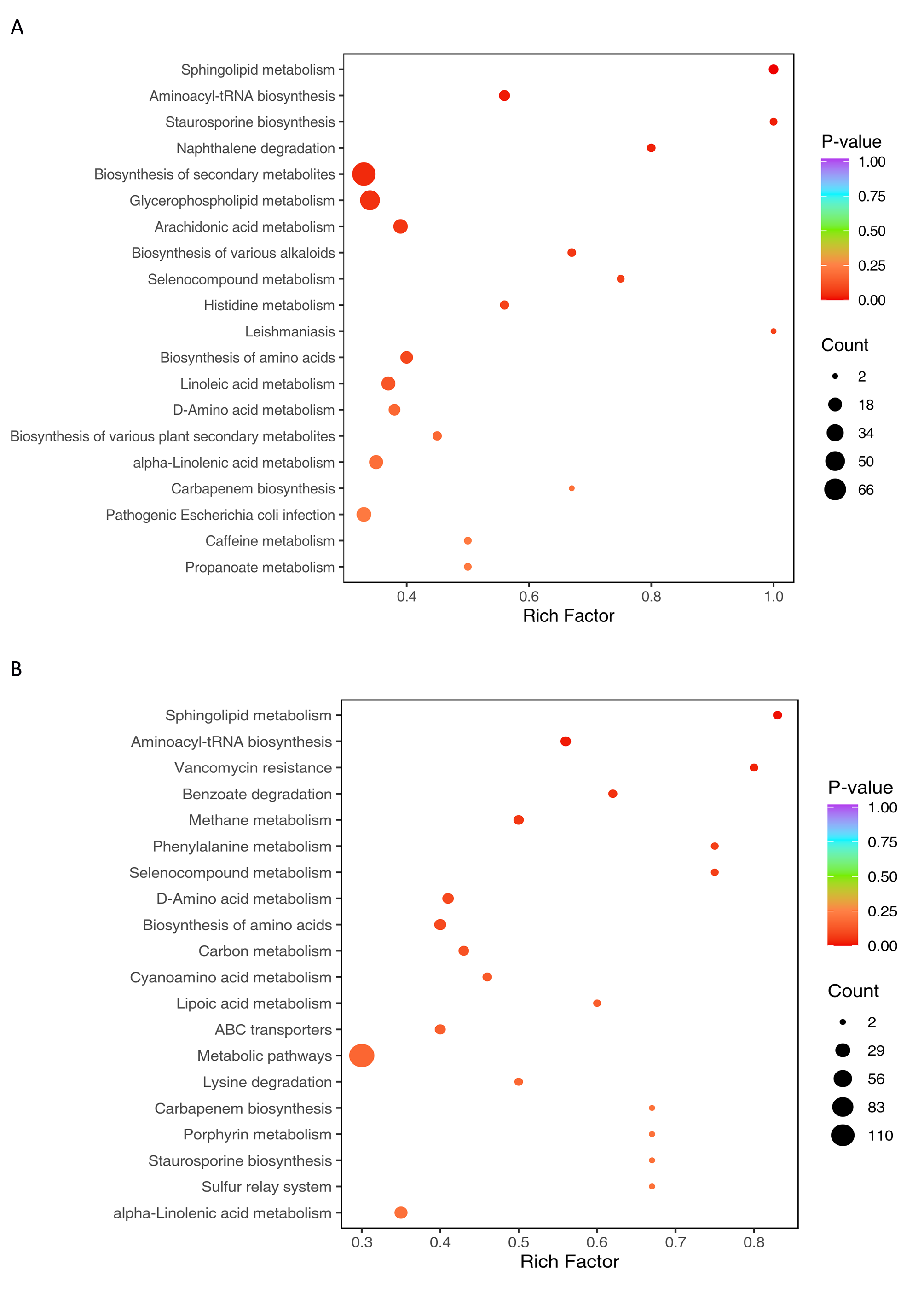

Supplement: Fig. S6 — Analysis data from multi-omics sequencing. [file mbio.00374-26-s0006.tif]

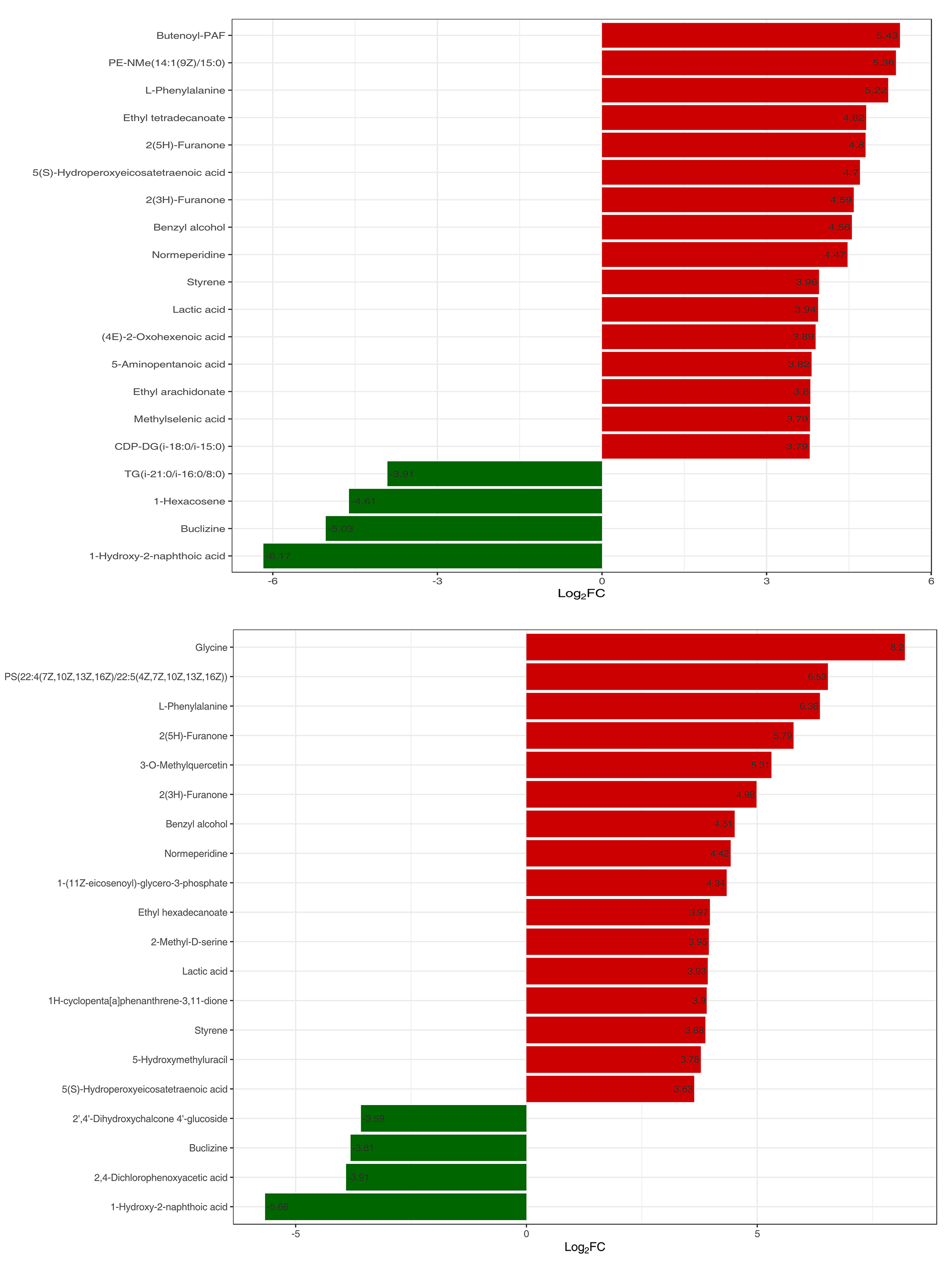

Supplement: Fig. S7 — Analysis data from multi-omics sequencing. [file mbio.00374-26-s0007.tif]
