# Supplementary material for: Prevotella bivia promotes cervical cancer progression and modulates macrophage polarization, while Lactobacillus iners suppresses these processes: evidence from multiomics analysis
Source: mBio. 2026 Jun 15;17(7):e00374-26. doi: 10.1128/mbio.00374-26 (PMC13344011; doi:10.1128/mbio.00374-26)
Supplement: Legends — for supplemental items. [file mbio.00374-26-s0008.docx]

**Supplementary Figure 1**

**Alpha and beta diversity analysis of the microflora in cervical tissue.**

(A-F) Alpha diversity indices, including Chao1, dominance, good coverage, observed features, Shannon and Simpson, in the 3 groups according to the results of the Tukey and Kruskal‒Wallis rank sum tests (C, Np, Nc). (G) Unweighted UniFrac heatmap of the distance matrix. In the same square, the upper and lower values represent the weighted UniFrac and unweighted UniFrac distances, respectively. (H, I) Beta diversity index including unweighted UniFrac and weighted UniFrac using the Tukey and Kruskal‒Wallis rank sum tests in the 3 groups. (J-L) NMDS analysis based on the Bray–Curtis distance, unweighted UniFrac distance and weighted UniFrac distance. NMDS, nonmetric multidimensional scaling.

**Supplementary Figure 2**

Kaplan-Meier PFS and OS curves of cervical cancer patients in the high-*Lactobacillus* abundance group compared with those in the low-*Lactobacillus* abundance group according to the log-rank test

**Supplementary Figure 3**

HeLa cells were subcutaneously injected into nude mice, followed by intraperitoneal administration of conditioned medium or PBS every other day. After 30 days, the mice were sacrificed.

(A) Lung tissues were collected for HE staining to assess metastatic tumor nodules.

(B) Representative lung CT scans from the three experimental groups.

(C) Three-dimensional reconstructed images of the lungs from each group.

**Supplementary Figure 4**

**Metabolomic sequencing of samples from patients with high- and low-abundance *Lactobacillus inners*/*Prevotella bivia***

(A) PLSDA of the high-abundance *Lactobacillus inners* tumour tissue and low-abundance *Lactobacillus inners* tumour tissue. (B) Volcano graph of the enrichment of all the metabolites in the two groups from A. (C) PLSDA of the high-abundance *Prevotella bivia* tumour tissue and low-abundance *Prevotella bivia* tumour tissue. (D) Volcano plots of the enrichment of all the metabolites in the two groups in C. (E, F) Matchstick map of differentially abundant metabolites in the comparisons from A and C, respectively. (G, H) KEGG enrichment bubble diagram for the comparisons from A and C.

**Supplementary Figure 5**

**KEGG classification of the differentially abundant metabolites in samples with high- and low-abundance *Lactobacillus inners*/*Prevotella bivia***

1. KEGG enrichment analysis of the high-abundance *Lactobacillus inners* tumour tissue and low-abundance *Lactobacillus inners* tumour tissue.

(B) KEGG enrichment analysis of the high-abundance *Prevotella bivia* tumour tissue and low-abundance *Prevotella bivia* tumour tissue.

**Supplementary Figure 6**

**KEGG analysis of the results of the metabolomic sequencing of the *Lactobacillus inners*/*Prevotella bivia* supernatant**

(A) The *Lactobacillus inners* supernatant vs. the PBS KEGG enrichment bubble diagram. (B) *Prevotella bivia* supernatant vs. PBS KEGG enrichment bubble diagram.

**Supplementary Figure 7**

**Metabolites whose abundance greatly changed in the *Lactobacillus inners*/*Prevotella bivia* supernatant.**

(A) The *Lactobacillus inners* supernatant vs. the PBS group. (B) The *Prevotella bivia* supernatant vs. the PBS group.

**Supplementary Table 1**

qPCR primer names and sequences.

**Supplementary Table 2**

16S rRNA Data.

**Supplementary Table 3-15**

Analysis data from multi-omics sequencing(S3-S12);software packages and algorithms(S13);clinical variables and lifestyle factors(S14-15).
